# Application of anatomic reconstruction technique for periurethral structure in robotic assisted laparoscopic radical prostatectomy

**DOI:** 10.3389/fonc.2023.1221217

**Published:** 2023-07-25

**Authors:** Haichang Li, Dongning Lu, Yuning Hu, Yixuan Mou, Dahong Zhang, Zhenghong Liu

**Affiliations:** Urology & Nephrology Center, Department of Urology, Zhejiang Provincial People’s Hospital (Affiliated People’s Hospital, Hangzhou Medical College), Hangzhou, Zhejiang, China

**Keywords:** robotic assisted laparoscopic surgery, radical prostatectomy, incontinence, enhanced recovery after surgery, anatomic reconstruction

## Abstract

**Objective:**

To investigate the outcome of patients underwent anatomic periurethral reconstruction during robotic assisted laparoscopic radical prostatectomy (RARP).

**Materials and methods:**

During August 2016 to May 2018, periurethral structure anatomic reconstruction was performed during RARP in 58 consecutive patients. The control group consists of another 50 patients had no reconstruction procedure during RARP. Perioperative data of these patients were collected retrospectively, including operation time, anastomosis time, intraoperative blood loss, duration of indwelling catheter, length of hospital stay, complications, postoperative pathology, and continence outcome at 1,3,6 and 12 months.

**Results:**

All cases were successfully performed without conversion to open or laparoscopic surgery. There were no major intraoperative or postoperative complications.The percentage of patients maintain continence in the reconstruction group versus non-reconstruction group: At 1 month 84.5% (49/58)versus 70.0% (35/50), at 3 months 89.7% (52/58)versus 78.0% (39/50), at 6 months 91.3% (53/58)versus 86.0% (43/50) and 1 year after surgery 100.0% (58/58)versus 96.0% (48/50). Reconstruction group showed better continence outcome in 1 and 3 months (P<0.05). There is no statistical differences in 6 month and 1 year.

**Conclusion:**

Anatomic reconstruction of periurethral structure during RARP is safe and feasible with reduced duration of indwelling catheter and better continence outcome.

## 1 Introduction

Prostate cancer is one of the most common malignant tumors in men. The incidence of prostate cancer is gradually increasing, and its incidence is the forth among malignant tumors in the world, with a dynamic growth (1). Radical prostatectomy is one of the most effective methods for localized prostate cancer. However, due to the serious impact of postoperative urinary incontinence on the life quality of patients (2), how to improve postoperative urinary control has been a research hotspot in urology for a long time. The PUI(Post-operative urinary incontinence) recovery rates were 5.7%, 23.4%, 64.6%, and 93.3% at 30, 90, 180, and 365 days following RARP (3). At present, numerous researches are devoted to the reconstruction of urine control tissue structure during operation, and have made some achievements, but in fact, these researches often only focus on reconstructing part of the structure of the anterior or posterior wall (4). Based on these structures of researches, we have firstly used anatomic reconstructed technique for periurethral structures in robotic-assisted laparoscopic radical prostatectomy.This study retrospectively analyzed the data of patients treated with this technique, compared it with patients treated with traditional urethrovesical anastomosis at the same time.The aim is to explore the safety and effectiveness of the anatomic reconstructed technique for periurethral structures, and to provide guidance and reference for the RARP.

## 2 Patients and methods

### 2.1 The demographic data

The admission criteria of this study were as follows:: (1) All patients were diagnosed with prostate cancer by transperineal prostate biopsy before surgery, and the PSA ≤ 20 ng/mL, Gleason score ≤ 7, clinical stage ≤ T2c; (2) Preoperative pelvic CT (Computed Tomography) and MRI (Magnetic Resonance Imaging) showed no periprostatic tumor invasion and pelvic lymph node metastasis, and the ECT (Emission Computed Tomography)/PET (Positron Emission Computed Tomography) showed no bone metastasis; (3) Life expectancy ≥ 10 years; (4) Transperineal prostate biopsy was performed at least 7 days before RARP; (5) Good physical condition, no communication barriers. Exclusion criteria: (1) patients with severe cardiopulmonary dysfunction; (2) Patients with severe bleeding tendency or coagulation dysfunction; (3) Patients who do not cooperate with treatment and cannot be followed up for a long time; (4) Diagnosis of prostate cancer after transurethral resection of prostate; (5) Patients with history of abdominal and groin surgery; (6) Patients with mental illness.A total of 108 cases were included, of which 58 cases were treated with anatomical reconstruction of peri-urethral structures (reconstruction group) and 50 cases with routine anastomosis (routine group).Before operation in both groups, urinary incontinence was excluded by inquiry of medical history, IPSS score scale or urodynamic examination. All operations were performed by the same surgeon with great robotic surgical skills (robot radical prostatectomy >200 cases).

### 2.2 Surgical method

The four-arm DaVinci Si (Intuitive Surgical, Sunnyvale CA, USA) Surgical System and a transperitoneal five-port Approach was used for all cases. The brief surgical presentation is presented in the **Supplementary Material**.

#### 2.2.1 Radical prostatectomy

The detailed surgical procedure are as follow: ① Open the peritoneum at the top of the bladder, blunt dissection the connective tissue in the extraperitoneal space of the bladder to the retropubic space, and remove the fat tissue overlying the prostate. ② Pay attention to persevering the bladder sphincter while the bladder neck is dissected using hot shears with an anterior approach, then lift the prostate, separate the seminal vesicles on both sides, and clip the vas deferens with a Hem-o-lock clip before disconnecting them. ③ Open the outer fascia layer of the Denonvillier fascia, along the outer surface of the Denonvillier fascia, close to the prostate and separate the prostate side ligament with shear (using an athermal technique), and continue to cut the remaining tissues along the prostate surface to the tip of the prostate with cold shear. When separating, pay attention to the use of Hem-o-lock clips to clip and then disconnect, and try not to use bipolar electrocoagulation. ④ At the attachment of pubic suspensory ligament of ventral prostate, dorsal venous complex (DVC) is not sutured. ⑤ Separate the DVC from the prostate surface, and then suture the broken edge to stop bleeding. ⑥ Seperated the tip of the prostate, cut off the urethra, pay attention to retaining sufficient length of urethral tissue, and use barbed suture to suture for hemostasis.

#### 2.2.2 Urethrovesical anastomosis

**Figure 1:**
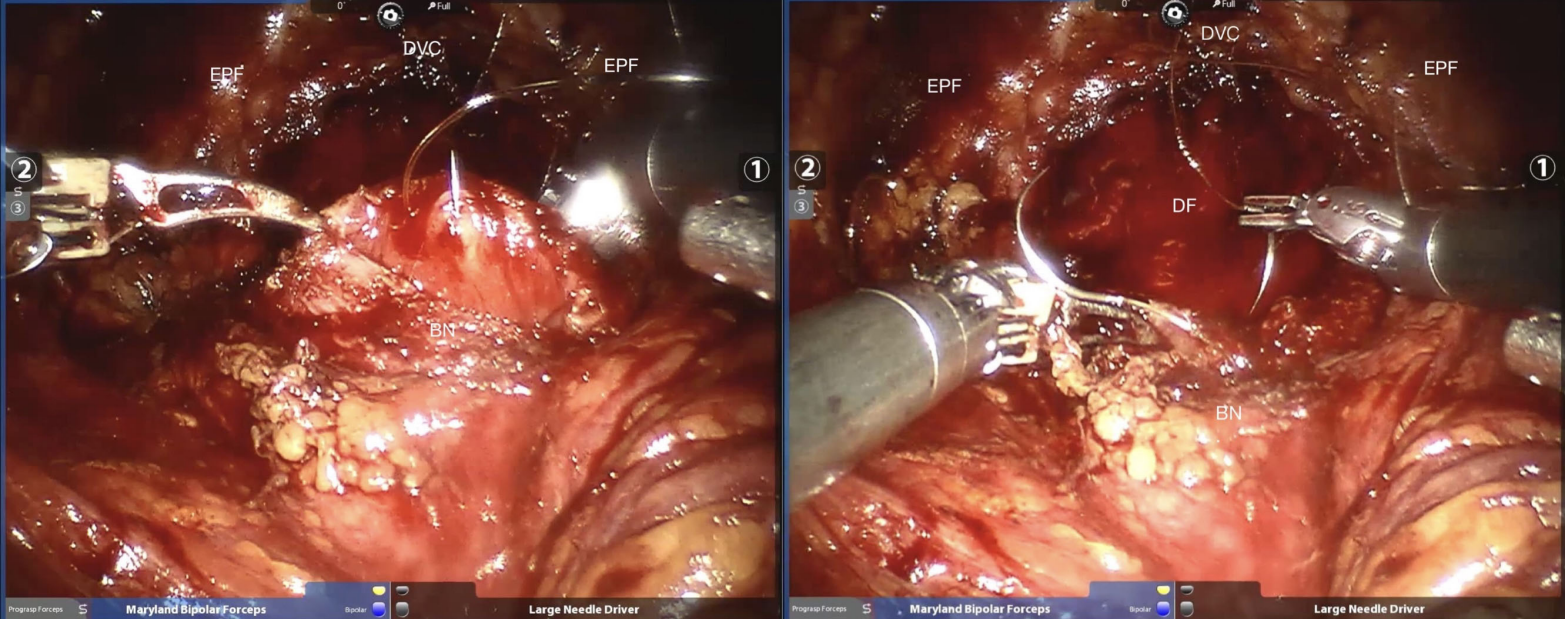
The needles were inserted at the location of six o’clock. BN, bladder neck; DF, Denonvillier fascia; DVC, dorsal venous complex; EPF, endopelvic fascia.

**Figure 2:**
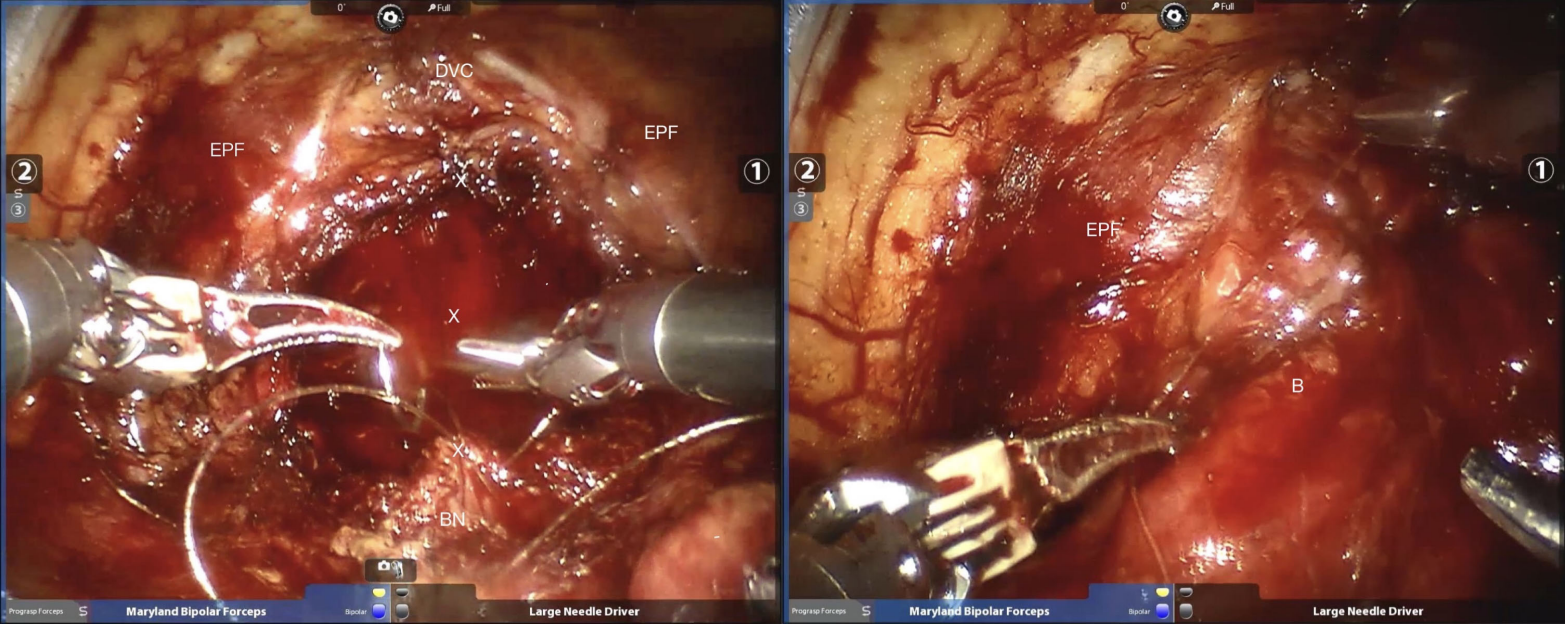
Strengthen the posterior wall of the urethra as well as pelvic floor fascia reconstruction. BN, bladder neck; x =stitch; DVC, dorsal venous complex; EPF, endopelvic fascia; B=bladder.

**Figure 3:**
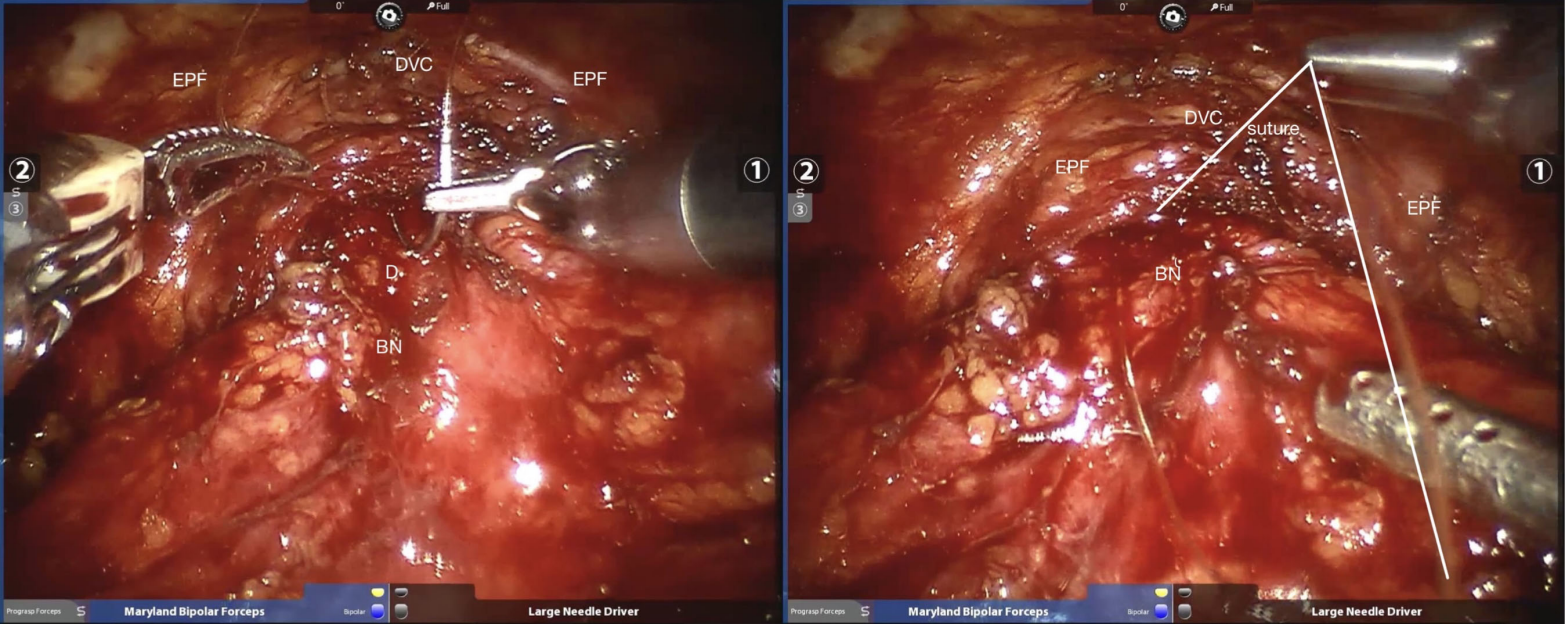
Sutured pubic prostatic ligament with the anterior wall of bladder neck. BN, bladder neck; D, detrusor; DVC, dorsal venous complex; EPF, endopelvic fascia.

The detailed surgical procedure are as follow: ① In the reconstruction group, the bladder and urethra were sutured continuously at 6 o’clock direction along the clockwise and counterclockwise with 3-0 barbed sutures and two needles (**Figure 1**). When suturing the urethra, a thin layer of Denonvillier fascia and connective tissue about 1 cm around the posterior edge of the urethral end shall be sutured into the urethra (**Figure 2**), and at the same time, the align of anastomosis between mucous membranes shall be ensured. ②We knot and suture at 12 o’clock to close other incisions of bladder. Besides, 3-0 barbed suture was used to suture the pubic prostatic skirt (pubic prostatic ligament, pelvic floor fascia) and the anterior wall of bladder neck which called detrusor apron, thus restore the anterior anatomical structure of urethra (**Figure 3**).

In the routine group, only double needle 3-0 barbed sutures were used, and the bladder and urethra were continuously sutured clockwise and anticlockwise from 6 o’clock, knotted at 12 o’clock and sutured to close the rest incisions in bladder.

#### 2.2.3 Bladder affusion

After the anastomosis, the bladder injection test was performed. Insert F18~20 rubber catheter through urethra, and inject 100~150mL normal saline to observe whether there is leakage at the anastomosis. If there is no obvious leakage, confirm that the suture is reliable, place a drainage tube behind the pubic bone, and close the incision.

### 2.3 Postoperative treatment and follow-up

Retrograde cystography was performed in all patients 7 days after operation to check vesicourethral anastomotic fistula. If there was no obvious anastomotic fistula, the catheter will be removed on the second day. In addition, the catheter was prolonged for 3 days in patients with anastomotic fistula, and the catheter was removed after the second retrograde cystography confirmed that there was no obvious anastomotic fistula.

The patients were followed up in 1, 3, 6 and 12 months after operation. Evaluation criteria for postoperative continence function: patients were considered continent if they were pad free all the time, or 1 urine pad for preventive use. If they use more than one urine pad every day, they will be defined as incontinence. IPSS score was performed 12 months after operation. During the follow-up, patients with dysuria should be further examined by urinary flow rate, urethral dilatation or urethroscopy to determine whether there is urethral stricture.

### 2.4 Statistical method

SPSS 22.0 statistical software was used to process the data. The measurement data in accordance with the normal distribution is expressed by means ± SD, and the measurement data that do not conform to the normal distribution is expressed by the median (range). Student’s t test was used to compare the measurement data between the two groups, and Wilcoxon rank sum test was used to compare the median. The counting data were expressed by example or percentage (%), and the comparison between the two groups was tested by χ 2 test and exact probability method. The difference was statistically significant (P < 0.05).

## 3 Result

All the 108 operations in this study were completed successfully and none of them were converted to open or laparoscopic surgery. No patient underwent bilateral pelvic lymph node dissection during operation. 98 patients with moderate and low risk prostate cancer(PSA ≤ 20ng/mL, Gleason score ≤ 7, clinical stage ≤ T2b) underwent bilateral neurovascular preservation, and 10 patients with T2C preserved bilateral, unilateral or no neurovascular bundle depending on the preoperative MRI. The patients’ characteristics are summarized in **Table 1**. The distribution of age, Prostate volume, PSA, BMI, preoperative Gleason score, clinical stage were compared among the two groups. It is worth mentioning that there is no significant statistical difference in these data.

**Table 1:** Comparison of Demographic data (mean ± SD).

Project	Reconstruction group (n=58)	control group (n=50)	p value
Age (year)	(73.7 ± 7.3)	(71.6 ± 5.4)	P=0.288
Prostate volume (cc)	(40.9 ± 5.8)	(39.8 ± 3.1)	P=0.390
Prostate specific antigen(PSA) (ng/mL)	(11.7 ± 2.4)	(13.2 ± 3.5)	P=0.165
Body Mass Index(BMI: kg/m2)	(24.2 ± 4.2)	(26.3 ± 3.2)	P=0.230
preoperative Gleason score n(%)			P=0.132
6 points	13(22.4)	10(20)	
7 points	45(77.6)	40(80)	
clinical stage n(%)			P=0.249
T1c	17(29.3)	13(26)	
T2a∼T2b	35(60.3)	33(66)	
T2c	6(10.4)	4(8)	

As for the intraoperative situation in **Table 2**, the operation time in the reconstruction group and the traditional group was 145.3 ± 12.3 min and 122.4 ± 11.4 min(P=0.023).Besides,the anastomosis time in the reconstruction group and the traditional group was 31.6 ± 8.2 min and 21.2 ± 4.4 min(P=0.011).Obviously, the reconstruction group took more time than the traditional group.In contrast, there was no statistical difference in intraoperative bleeding volume between the two groups(P=0.095),108.1 ± 8.3mL in the reconstruction group and 103.3 ± 10.4 in the traditional group.The 4 patients in two groups (P=0.063)with positive bladder injection test did not find any obvious leakage in the operation. Only water exuded from around the reconstructed urethra. The suspected leakage was reinforced with 3-0 Vicryl and then the water injection test was performed. The anastomosis was ended after the negative water injection test. No obvious urine leakage was found after the operation

**Table 2:** Comparison of perioperative data between two groups of patients undergoing robotic radical prostatectomy (mean ± SD).

Project	Reconstruction group (n=58)	control group (n=50)	P value
Operation time (min)	145.3 ± 12.3	122.4 ± 11.4	0.023
Anastomotic time (min)	31.6 ± 8.2	21.2 ± 4.4	0.011
Intraoperative bleeding volume (mL)	108.1 ± 8.3	103.3 ± 10.4	0.095
Failure of water injection test n(%)	1 (1.7)	3 (4.0)	0.063
Time of indwelling catheter after operation (d)	7.0 ± 0.5	11.0 ± 0.6	0.028
Time of removal of drainage tube after operation (d)	3.0 ± 1.2	4.0 ± 1.6	0.342
Postoperative hospital stay (d)	8.0 ± 1.1	10.0 ± 1.5	0.142
Post operative complication n(%)	4 (6.9)	4 (8.0)	0.186
Pathological stage n(%)			0.252
pT2a∼T2b	53 (91.4)	46(92.0)	
pT2c	5 (8.6)	4(8.0)	
Postoperative Gleason score n(%)			0.067
6 points	19 (32.8)	15 (30.0)	
7 points	30 (51.7)	25 (50.0)	
8 points	9 (15.5)	10 (20.0)	
Positive margin n(%)	3 (5.2)	2 (4.0)	0.129
Postoperative continence rate n (%)			
At 1 mo	49 (84.5)	35 (70)	P=0.029
At 3 mo	52 (89.7)	39 (78)	P=0.047
At 6 mo	53 (91.3)	43 (86)	P=0.079
At 12 mo	58 (100)	48 (96)	P=0.146
Type of complications n (%)			
incisional hernia	1 (1.72)	0 (0.0)	P=0.321
inguinal hernia	1 (1.72)	2 (4.0)	P=0.483
incomplete intestinal obstruction	1 (1.72)	0 (0.0)	P=0.321
abdominal infection	1 (1.72)	0 (0.0)	P=0.321
ankylo-urethria	0 (0.0)	2 (4.0)	P=0.123
IPSS score	10.4 ± 1.6	12.1 ± 1.3	P=0.367

**Figure 4:**
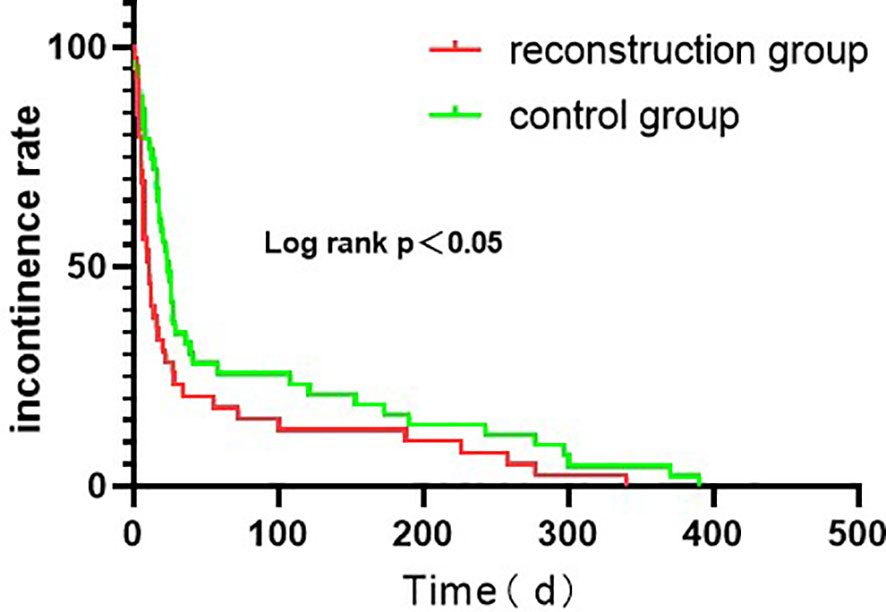
Kaplan–Meier curve showing incontinence reduction rate over Time.

As for Postoperative status,the postoperative indwelling catheter time in the reconstruction group was shorter than that in the control group (P = 0.028). There was no significant difference in the time of removal of drainage tube, postoperative complications and postoperative hospital stay between the two groups (P > 0.05). Postoperative complications occurred in 4 cases in the reconstruction group, including incisional hernia, inguinal hernia, incomplete intestinal obstruction and abdominal infection. However, Postoperative complications occurred in 4 cases in the routine group, including 2 cases of inguinal hernia and 2 cases of urethral stricture. The patients with incisional hernia and inguinal hernia underwent laparoscopic herniorrhaphy. The patients with incomplete intestinal obstruction after operation were ordered to fast, indwelling gastric tube, giving intravenous nutrition treatment, and resuming eating after intestinal function improvement. The patients with abdominal infection were treated with sensitive antibiotics. In addition, these patients with the above complications recovered and had good results after related treatment. In the control group, 2 cases had progressive dysuria and were diagnosed as urethral stricture by cystoscopy. Regular outpatient urethral dilatation was given once a week and improved after 2 months.2 cases were followed up for 6 months after urethral dilatation, and there was no recurrence of urethral stricture. Hence there was no significant difference in the incidence of urethral stricture between the two groups (P > 0.05). The continent rates in the reconstruction group were 84.5% and 89.7% in 1 and 3 months after operation, respectively, which were better than 70.0% and 78.0% in the control group, and the difference was statistically significant (P < 0.05). In 6 and 12 months after surgery, continent rates were 91.3% and 100.0% in the reconstruction group, and 86.0% and 96.0% in the control group, respectively. There was no statistical significance between the two groups (P > 0.05). We have analyzed the urinary incontinence rate with Kaplan - Meier analysis (**Figure 4**). The IPSS scores of the reconstruction group and the control group were 10.4 ± 1.6 and 12.1 ± 1.3, respectively, with no statistical significance (P > 0.05). The pathological stages of 108 patients were pT2a ~ pT2c, which was consistent with the preoperative clinical stage. No prostate capsule or seminal vesicle invasion was found in all patients. There was no significant difference in postoperative pathological stage, postoperative Gleason score and rate of positive incisal margin between the two groups. Patients with positive incisal margin were given adjuvant endocrine therapy 6 weeks after operation, and radiotherapy within 3 to 6 months after operation. All patients were followed up for 12 months and there was no biochemical recurrence.

## 4 Discussion

Laparoscopic radical prostatectomy is the standard procedure for the treatment of early localized prostate cancer. However, it inevitably causes great damage to the patient’s anatomical structure, so that patients often need to indwelling catheter for a long time after surgery (5). This may bring complications to patients, including bladder spasm, bacteriuria, urinary tract infection, pyelonephritis, urinary tract injury, catheter obstruction, bacteremia, etc (6). Remarkably, studies have reported that after indwelling catheter for more than 1 week, the incidence of urethral strictures will increase (7). Hence, reducing the time of indwelling catheter after RARP is crucial for patient prognosis and subsequent recovery.

Notably, urinary incontinence after radical prostatectomy has always been one of the focuses of surgical research on prostate cancer. In addition to the patient’s own reasons (age, obesity, hypoactivity, basic vesico-urethral functional status) (8–11), the recovery of postoperative urinary control is related to the preservation of bladder neck, residual urethral length, injury of urethral sphincter, protection of nerve vascular bundle and other factors, and the exact mechanism is still not completely clear (12). Yet, it is certain that good urinary control is the result of the combined effect of the recovery of the complete functional structure and the preservation of the vascular nerve bundle. Current research results demonstrate that the long-term urine control effect is ideal after radical prostatectomy, and the urine control recovery rate can reach 85% ~ 97% in 12 months after surgery, but the early postoperative urine control recovery effect is not satisfactory (13). A large number of studies have been innovated and explored to achieve early postoperative recovery of urinary control (14–18). At present, many medical centers at home and abroad have adopted various surgical techniques to improve postoperative urinary control, and achieved certain clinical effects (19–21). Studies have shown that retain or repair of tissue structures related to urinary continence as far as possible is the best measure to achieve early recovery (22).

The periurethral anatomical reconstruction technique used in this study is to repair the urethral and the periurethral structures without damaging the Retzius space and opening the pelvic fascia. The following aspects should be paid attention to in the surgical techniques:1. When the posterior lip of the urethra is anastomosed, the thin layer of denonvillier fascia and connective tissue about 1cm around the posterior edge of the urethra is sutured to strengthen the posterior wall of the urethra, reduce the anastomotic tension of the urethra, and prevent urine leakage. It’s worth noting that this method can achieve non-dead cavity closure of the posterior urethra, reduce the formation of surrounding bleeding and hematoma, and promote healing. 2. After the urethra was anastomosed at the position of 12 o’clock and the rest of the bladder incisions closed, the pubic prostatic skirt (pubic prostatic ligament, pelvic floor fascia) was sutured with the anterior wall of the bladder neck, namely the broken edge of the detrusor muscle, so as to restore the anterior urethral anatomy. This step is completed on the basis of not destroying the structures in the Retzius space (not opening the pelvic fascia and not sewing the DVC), which helps to protect the external urethral sphincter and nerves at the tip of the prostate. Then, suture the undamaged pubic prostate skirt (pelvic fascia, arc tendon ring, pubic pubic suspension ligament of prostate) to the anterior wall of bladder, anatomically restore the suspension structure in front of the prostate, reduce the tension of urethral bladder anastomosis, and restore the structural stability of the urethra and bladder, which is very helpful for the early recovery of postoperative urinary control. 3. The urethra and the bladder neck were anastomosed with double needles, that is, the bladder-urethra anastomosis was continuously sutured from the position of 6 o’clock to both sides with 3-0 double needles and ended at the position of 12 o’clock. During the suture process, attention should be paid to the anastomosis of urethra and bladder neck mucosa to prevent poor healing caused by serosa suture. In addition, double-needle suture can control the suture tension on both sides and make the whole anastomotic ring tightness evenly. Compared with single-needle suture, double-needle suture can reduce the phenomenon that the starting point of the anastomosis is too loose or the opposite side is too tight, and thus reduce the occurrence of postoperative urethral stricture or urine leakage.

Results from this group of the study show that the operation time and anastomosis time in the reconstruction group is slightly longer than those in the routine group, because it takes more time to repair the structures around the urethra, but the anastomosis time decreases with the increase of proficiency.In this study, there was no significant difference in intraoperative blood loss and postoperative complications, which reflects the high safety of anatomic reconstruction technique for periurethral structure. In terms of postoperative indexes, the time of indwelling catheter in the reconstruction group was significantly shorter than that in the routine group, which may be due to the strengthening of support and traction in the structures around the urethra in the reconstruction group, so that the tension of the urethral anastomosis was at its lowest station. Hence, it can achieve the effect of rapid recovery combined with early removal of urinary catheter, which can not only improve the comfort of patients, but also reduce urinary tract infection caused by indwelling catheter. The results of literature also indicate that the reconstruction of the posterior wall can reduce the occurrence of postoperative vesicourethral anastomotic fistula (23). Nevertheless, there are still some disadvantages because of the large suture tension or postoperative anal pain. Therefore, in this study, only suturing the tissue near the urethra instead of the traditional posterior wall suture can not only reduce tension, but also stop bleeding and close the posterior dead space of the urethra. The”Rocco Stitch” (24), well known to robotic surgeons, aims to reconstruct this posterior plate for enhanced urethral coaptation and closure.Reconstruction of the anterior urethral wall can stabilize the urethra and the urethra rod sphincter in a normal anatomical position, while providing support to the anterior wall of the urethra, Patel et al. (14) used anterior urethral wall suspension technique, which significantly improved the urine control rate of patients at 1 and 3 months after operation. This study combines the advantages of both and improves on this basis. The results of this study demonstrated that the reconstruction group was superior to the routine group in urine control 1 and 3 months after operation, indicating that the repair of periurethral structure could improve the early urine control recovery rate, which was similar to the results reported in domestic and foreign literature (14, 24). In the other hand, it’s worth mentioning that in this study, prostatectomy was performed by using a modified anterior approach to preserve the structure in the Retzius space (16) and thus, the suspension structure in front of the urethra was not greatly damaged, which was more consistent with the original anatomy than using sutures to restore this part of the suspension structure in the same type of study. In our study, except for the same resection methods,there was no significant difference in the tumor control between the two groups, it also indicates that the urethral reconstruction technique has reliable therapeutic effects. The indications for lymph node is section in our study were PSA > 20 ng/mL or Gleason score > 7 or clinical stage > T2c or imaging examination suggested pelvic lymph node metastasis.Pelvic lymph node dissection (PLND) is the “gold standard” to judge whether lymph node metastasis (LNM) occurs in prostate cancer patients. It may have a certain therapeutic effect as well. EAU guidelines, NCCN Guidelines, and AUA guidelines all recommend pelvic lymph node dissection in patients at high risk for LNM. Among them, EAU guidelines recommend intermediate-risk prostate cancer with LNM ≥ 5%; The NCCN guidelines recommend PLND for high-risk and very high-risk prostate cancer with an LNM ≥ 2%. However, after PLND, the complication rate and operation time and so on will increase. Therefore, we considered pelvic metastasis or lymph node metastasis as confounding factors that could cause biases. To sum up, we selected low - and intermediate-risk patients who did not necessarily need PLND. We compared the results of studies with anterior urethral wall suspension technique or Posterior musculofascial reconstruction alone with the results of this study, and found significant differences in urinary incontinence rates at 1 month and 3 months after surgery. The percentage of patients who had achieved continence in the anterior reconstruction group were 40%, 92.8%, 97.9% and 97.9% at the 1, 3, 6, 12 months follow-up (14), respectively. The percentage of patients who had achieved continence in the posterior reconstruction group were 52.3%, 86.5%, 92.3% and 92.3%, respectively (25). Our total reconstruction group had continence rates of 84.5%, 89.7%, 91.3%, and 100% at 1, 3, 6, and 12 months, respectively. From the perspective of postoperative urinary continence rate, the data disclosed in the previous studies compared with the data in our study, our reconstruction had higher urinary continence rate in 1 months and 3 months after operation, which means that Our reconstruction performed as well as previous studies in the long run, with better results in the early stages.

## 5 Conclusion

To sum up, the anatomical reconstruction technique of periurethral structure adopted in this study is safe and feasible, which can not only achieve the purpose of radical resection of tumor, but also remove the catheter in a short time after operation and restore urine control function early. However, the operation time in this study was significantly different from that in the control group, so it is necessary to simplify the operation process and become proficient in this technique. For intermediate-high risk prostate patients, pelvic lymph node dissection should also be added to the program to adapt to different clinical and pathological stages. The number of cases in this study is limited, and the findings need to be further confirmed in a multicenter prospective controlled study with more data.

## Data availability statement

The original contributions presented in the study are included in the article/**Supplementary Material**. Further inquiries can be directed to the corresponding authors.

## Ethics statement

All study participants were informed about the planned procedure and signed informed consent. The study was approved by the ethics committee of Zhejiang Provincial People’s Hospital, Hangzhou city, China (2020QT023).

## Author contributions

DZ, HL, and ZL: Project development. HL, and DL: Manuscript writing. YH, YM, and HL: Data collection and analysis. All authors contributed to the article and approved the submitted version.
